# Educational Case: Paget disease of bone

**DOI:** 10.1016/j.acpath.2026.100289

**Published:** 2026-08-24

**Authors:** James Lee, Jonathan J. Light, Prajeeth K. Koyada, Davsheen Bedi, Matthew R. DiCaprio

**Affiliations:** a Albany Medical College, Albany, NY, USA; b Department of Orthopaedic Surgery, Albany Medical Center, Albany, NY, USA; c Department of Pathology, Albany Medical Center, Albany, NY, USA; d Division of Orthopaedic Oncology, Albany Medical Center, Albany, NY, USA

**Keywords:** Pathology competencies, Organ system pathology, Musculoskeletal system, Non-neoplastic disorders, Paget disease of bone

The following fictional case is intended as a learning tool within the Pathology Competencies for Medical Education (PCME), a set of national standards for teaching pathology. These are divided into three basic competencies: Disease Mechanisms and Processes, Organ System Pathology, and Diagnostic Medicine and Therapeutic Pathology. For additional information, and a full list of learning objectives for all three competencies, see https://doi.org/10.1016/j.acpath.2023.100086 ^1^

## Primary objective

Objective MS2.6: Paget Disease. Discuss the clinicopathologic changes of Paget disease, including the histologic phases and complications of this disorder.

Competency 2: Organ System Pathology; Topic: Musculoskeletal System (MS); Learning Goal 2: Non-neoplastic Disorders of the Musculoskeletal System.

## Patient presentation

A 76-year-old woman presents to the clinic with left arm pain for the past three months. She states that the pain has been progressive and does not recall any trauma. She also notes a history of progressively worsening hearing loss over the past year. She has a history of right hip and knee arthroplasties. Her past medical history is significant for chronic sinusitis, diverticulitis, hyperlipidemia, and cervical spondylosis. She is currently taking metoprolol 100 mg daily, atorvastatin 20 mg nightly (tolerated for ten years without myalgias), omeprazole 20 mg every morning, levothyroxine 50 mcg daily, and fluticasone nasal spray PRN (*pro re nata*), i.e., as needed. Her last dose of alendronate was approximately three months ago. For her pain, she has increased her baseline use of oral ibuprofen over the past few months.

## Diagnostic findings, Part 1

The patient's vital signs are: blood pressure 139/75 mm Hg, heart rate of 72 beats per minute (bpm), respiratory rate of 16 breaths per min, temperature 98.4 °F, and blood oxygen saturation 95% on room air. Physical examination reveals that the patient appears well but is in mild distress. Cardiopulmonary exam reveals normal S1 and S2 with a regular rate and rhythm; there are no crackles or wheezing on lung auscultation. The left upper extremity demonstrates asymmetry and slight angulation compared to the right. Her left anterior shoulder and humerus are tender, without erythema, warmth, or fluctuance. Her active and passive range of motion, strength, and sensation in the left upper extremity are intact and equal to those of the contralateral side. Her bilateral lower extremity sensory and motor examination are intact.

The patient's progressive left upper arm pain with mild humeral angulation warrants radiographs of the humerus and shoulder as the next best step in diagnosing an underlying problem. Radiographs allow assessment of bone quality, the presence of current or prior fractures, callus formation, or underlying osseous lesion abnormalities attributed to metabolic bone disease. Radiographs are first-line diagnostic tools, particularly when extremity pain persists despite conservative therapy, as ongoing symptoms raise concern for an underlying osseous pathology. In atraumatic cases or when osseous pathology is suspected, additional laboratory testing such as markers of bone breakdown C-terminal telopeptide of type 1 collagen (CTX) and N-terminal telopeptide (NTx), and bone formation including bone-specific alkaline phosphatase (BSAP), alongside metabolic panels (including renal function, calcium, phosphate, and vitamin D levels) can be useful in narrowing the differential diagnosis.^2^

Specialized laboratory panels are generally reserved for suspected metabolic disturbance or atypical clinical presentations. Elevated bone formation and resorption laboratory results combined with radiographic evidence of osteoblastic and osteolytic lesions could suggest metastatic bone disease (MBD) or primary bone tumor as some of the possible diagnoses. This underscores the importance of approaching discussions with compassion and personalization given the potential psychological and social impact of the diagnosis.

## Questions/discussion points, Part 1

### Describe what you see on the left humerus radiographs and clinically correlate them with the laboratory results

**Fig. 1:**
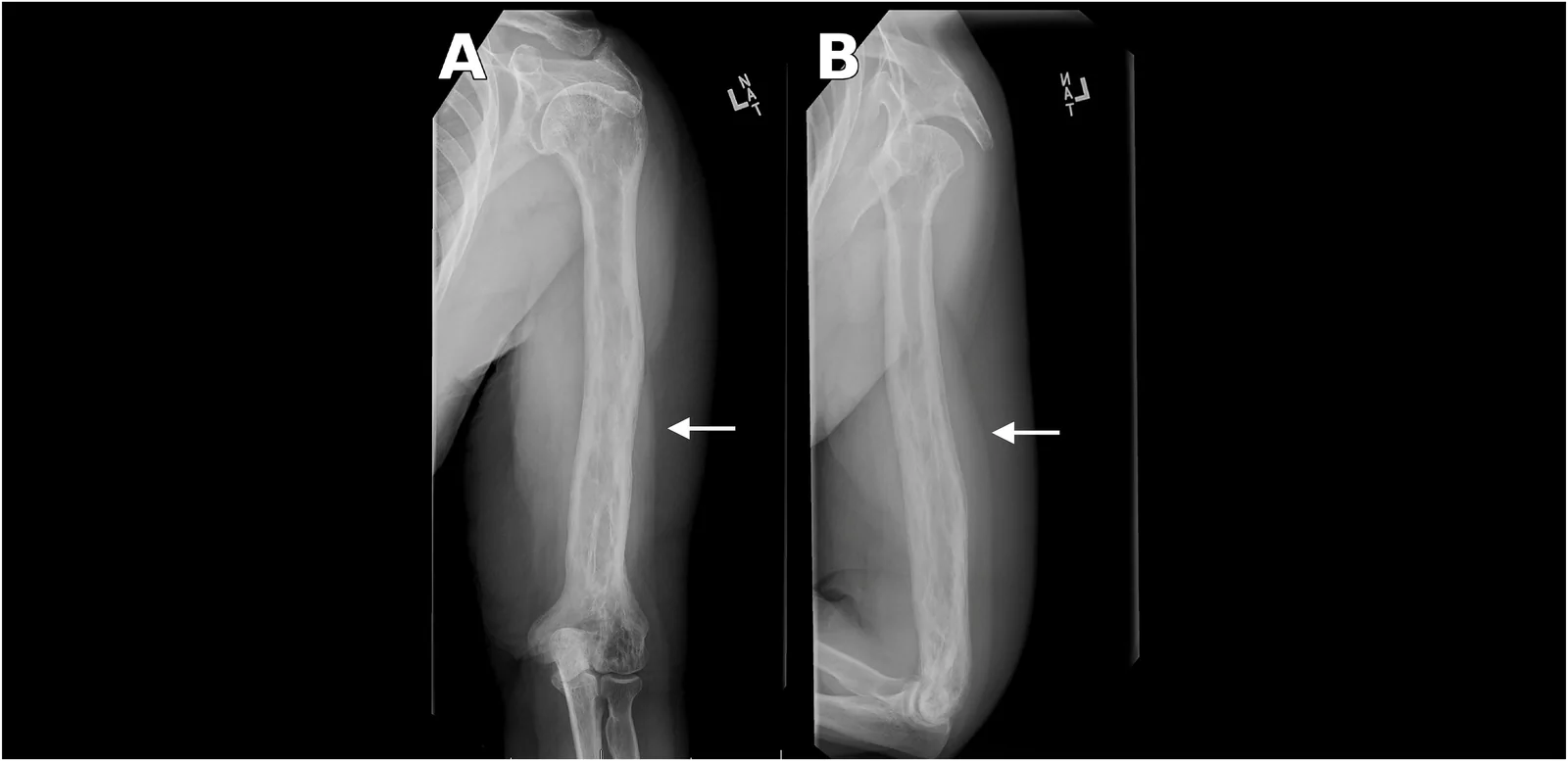
**(A)** Anteroposterior and **(B)** lateral radiographs of the left humerus demonstrating mild curvature (arrows) of the diaphysis, convex laterally, with cortical and trabecular thickening with increased width in the mid and distal diaphysis (bracket). Created in BioRender.com by JJL.

Anteroposterior and lateral radiographs of the left humerus demonstrate areas of bone destruction and repair with a coarse trabecular pattern, as well as areas of radiolucency mixed with areas of bone sclerosis or formation throughout the length of the humeral diaphysis and distal metaphysis. The diameter of the humerus is expanded compared to the normal contralateral arm. This occurs over an extended period of time because of increased resorption and bone formation. There is no fracture identified, although there is a slight varus and extension deformity of the humerus centered at the mid-diaphysis (Fig. 1). BSAP was found to be 75.2 mcg/L (normal 7.0–22.4 mcg/L).^2^ Collagen breakdown markers revealed elevated levels of urinary hydroxyproline of 80 mg/24 hr, NTx of 142 nM, and CTX of 1184 pg/mL (Table 1). Complete blood count (CBC), a routine laboratory order to assess the patient's general health, shows no leukocytosis expected with an infection and no sign of acute or chronic bleeding based on the hemoglobin and hematocrit (Table 2).

**Fig. 2:**
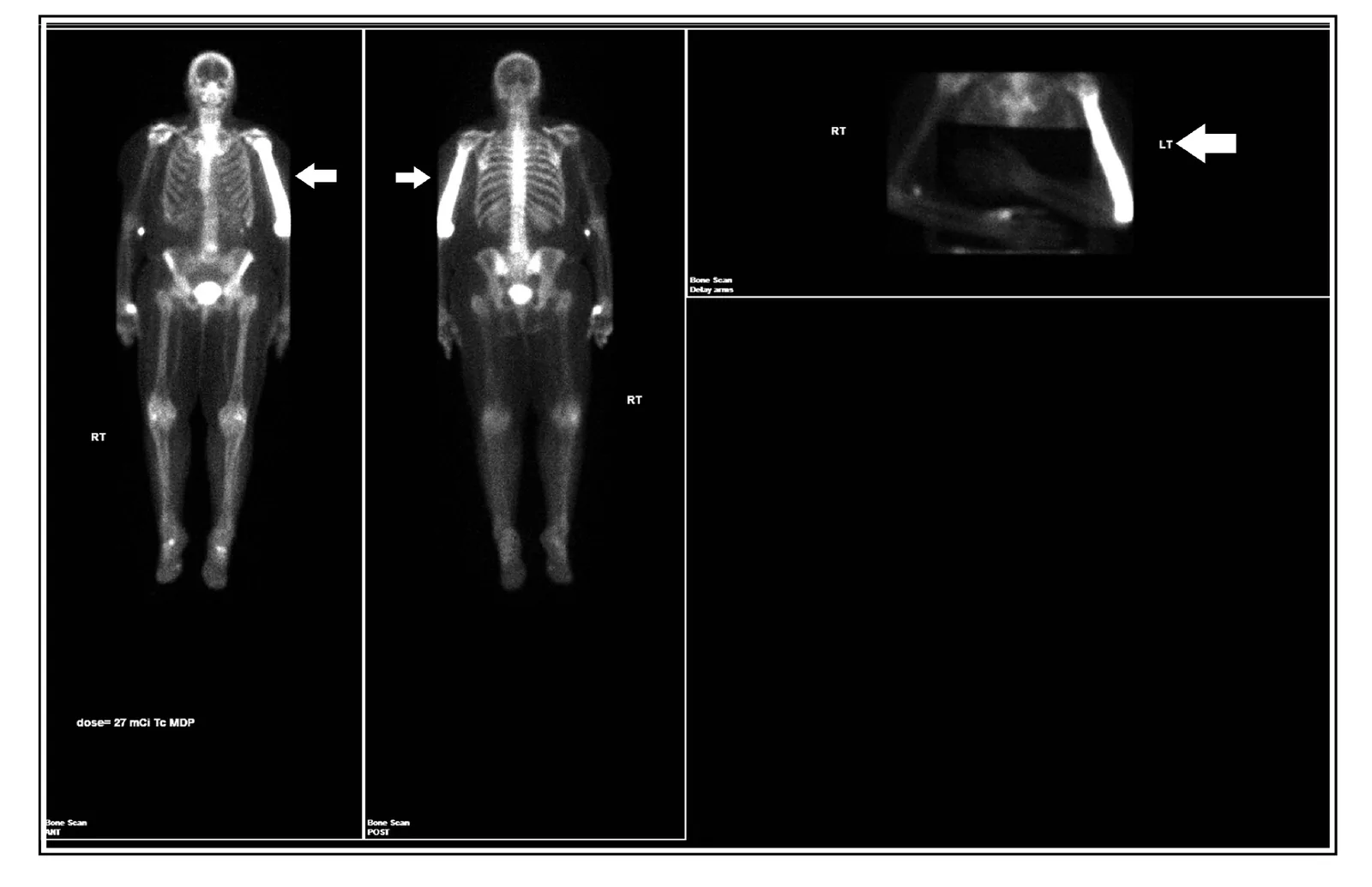
Bone scintigraphy shows marked increased activity throughout the entire left humerus (arrows), with mild increased activity in the L5 vertebral body. Created in BioRender.com by JJL.

**Table 1:** Chemistry profile.

Chemistry profile	Results	Reference range
Sodium, serum (mEq/L)	136	135–145
Potassium, serum (mEq/L)	4.5	3.5–5.0
Chloride, serum (mEq/L)	101	95–105
Carbon dioxide (mm Hg)	26	35–45
Anion gap (mEq/L)	9	4–12
Calcium, serum (mg/dL)	9.2	8.4–10.2
Blood urea nitrogen (mg/dL)	16	8–20
eGFR (mL/min)	90	90–120
Glucose (mg/dL)	93	70–100
Alkaline phosphatase (U/L)	161	30–120
Albumin (g/dL)	4.3	3.5–5.5
Total protein (g/dL)	7.1	5.5–9.0
Aspartate aminotransferase (AST) (U/L)	15	10–40
Alanine aminotransferase (ALT) (U/L)	11	10–40
Bilirubin, total (mg/dL)	0.4	0.3–1.0
Vitamin D, 25-hydroxy (ng/mL)	15.8	5–75
Bone specific alkaline phosphatase (mcg/L)	75.2	7.0–22.4
Urinary hydroxyproline (mg/24 hr)	80	10–30
N-Telopeptide (nM)	142	26–124
C-telopeptide (pg/mL)	1184	104–1008
Thyroxine (T4), free (ng/dL)	0.96	0.8–1.8
Thyroid stimulating hormone (TSH) [μU/mL]	5.970	0.5–4.0

**Table 2:** Complete blood count.

Complete blood count	Results	Reference range
WBC (10^9^/L)	6.4	4.5–11.0
RBC (million/mm^3^)	4.43	3.5–5.5
Hemoglobin (g/dL)	12.3	12.0–16.0
Hematocrit (%)	37.6	37–47
Platelet count (/μL)	330	150–400
MCV (μm^3^)	84.9	80–100
MCH (pg)	27.8	28–32
MCHC (g/dL)	32.7	33–36
MPV (μm^3^)	9.6	7–12
RDW-SD (μm^3^)	42.7	40.0–55.0

RBC: red blood cell; WBC: white blood cell; MCV: mean corpuscular volume; MCH: mean corpuscular hemoglobin; MCHC: mean corpuscular hemoglobin concentration; MPV: mean platelet volume; RDW-SD: red cell distribution width, standard deviation.

### What is your differential diagnosis based on the clinical history, radiographic findings, and laboratory findings?

The patient's progressive, non-traumatic, localized arm pain with radiographic findings of coarse trabeculation and mild bony expansion raises concern for several potential pathologic processes affecting the humerus. In an older patient (65+ years), the major considerations include neoplastic, infectious, and metabolic bone disorders, each of which can produce the chronic pain and alteration in bone architecture. The three most clinically relevant diagnoses in this context are metastatic carcinoma, long-standing chronic osteomyelitis, and Paget disease of bone. Understanding each is critical to directing appropriate diagnostic workup and management.

Metastatic carcinoma is the most common cause of destructive bone lesions in patients >40 years of age and should be strongly considered when accompanied by progressive bone pain and radiolucent and/or sclerotic changes on radiographs. Bone metastases originate most frequently from breast, prostate, lung, kidney, and thyroid carcinomas.^3^ Lesions may be purely radiolucent, sclerotic, or mixed, depending on the primary tumor biology.^3^ The tumor often presents as poorly marginated, medullary-based areas of destruction with cortical breakthrough.^3^ While multifocal involvement is typical, unifocal involvement does not exclude metastatic carcinoma. The disease tends to favor the axial skeleton and proximal long bones, including the humerus.^3^ Patients may report night pain, functional decline, or symptoms related to the primary malignancy.^3^

Radiographically, metastatic carcinoma may lack the uniform cortical thickening or bony expansion seen in Paget disease.^4^ Also, lesions related to metastatic carcinoma to bone typically affect regions of bone and rarely involve the entire diaphysis of an involved bone. Laboratory findings may include elevated alkaline phosphatase (ALP) in osteoblastic metastases, but inflammatory markers are usually normal unless a fracture or superimposed infection is present.^3^

Chronic osteomyelitis is an infection of bone in which patients typically present with localized bone pain, tenderness, and occasionally swelling. Systemic symptoms are often absent in chronic osteomyelitis.^5^ Radiographically, chronic osteomyelitis classically demonstrates a mixture of radiolucent destruction and reactive sclerosis, sometimes with cortical thickening from periosteal new bone formation.^6^ Sequestrum and involucrum are hallmark features of osteomyelitis.^6^ In adults, the most common sites for chronic osteomyelitis include the diaphysis of long bones, the tibia being the most common location related to prior injury and surgical treatment and limited soft tissue wrapping of the bone, and the thoracic and lumbar vertebral bodies.^7^

Chronic osteomyelitis can mimic malignancy on imaging, but the presence of a draining sinus tract, regional skin changes, or significantly elevated ESR (erythrocyte sedimentation rate)/CRP (c-reactive protein) increases suspicion for infection. Osteomyelitis often affects a single bone in a localized pattern and may show periosteal reaction associated with chronic inflammatory remodeling.^6^

Clinically, Paget disease of bone often presents incidentally, but when symptomatic, the most common complaint is persistent, localized bone pain that is dull and aching in character.^8^ The condition usually affects adults over age 50 and commonly involves the pelvis, femur, spine, skull, and humerus.^8^ Neurologic symptoms include hearing loss with skull involvement and radiculopathy with spinal involvement due to bony overgrowth.^9^ Paget disease typically lacks systemic inflammatory symptoms, which helps differentiate it from chronic osteomyelitis or malignancy presenting with constitutional symptoms.

From an imaging standpoint, radiographs are the cornerstone of diagnosis. Paget disease radiographs classically demonstrate a combination of cortical thickening, coarse trabeculation, and bony expansion.^9^ These findings typically involve a majority of the long bone involved or the entire innominate bone (hemipelvis). Computed tomography (CT) further delineates cortical thickening and deformity, while bone scintigraphy is highly sensitive for identifying additional sites of involvement and assessing disease activity through increased radiotracer uptake.^10^

Laboratory evaluation supports the diagnosis by demonstrating elevated bone turnover markers, with serum ALP being the most widely used indicator for active disease.^11^ ALP is elevated in most untreated or progressive cases. Markers of collagen breakdown such as urinary hydroxyproline, N-terminal telopeptide, and C-terminal telopeptide, may be elevated.^11^ Importantly, serum calcium, phosphate, and inflammatory markers (ESR/CRP) are generally normal, which helps distinguish Paget disease from chronic infection or metastatic malignancy.^12^

### What is the workup for suspected Paget disease of bone, and what do the diagnostic results support as the diagnosis?

Patients presenting with idiopathic serum ALP elevation, incidental radiographic findings of bone disease, or clinical features such as bone pain, tenderness, or bowing, raise the index of suspicion for Paget disease of bone.^8^

The workup for Paget disease of bone involves a combination of clinical assessment, laboratory tests, and imaging studies to confirm the diagnosis and evaluate the extent of the disease. During clinical evaluation, some common symptoms that increase suspicion for Paget disease include persistent bone pain, bone deformities, hearing loss, cardiac failure (secondary to increased vascularity of pagetoid bone), history of fractures, and joint pain.^8^

Laboratory tests ordered for continued evaluation include serum ALP, serum calcium and phosphorus, and parathyroid hormone (PTH). In Paget disease, serum ALP is typically elevated due to increased osteoblastic activity. Serum calcium and phosphorus levels are usually normal unless there are complications, such as immobilization leading to hypercalcemia. Parathyroid hormone levels are obtained to rule out familial hyperparathyroidism as part of the differential diagnosis.^13^ In addition, part of every lab workup for possible Paget disease of bone is urinary hydroxyproline, N-terminal telopeptide, and C-terminal telopeptide, which are markers of bone turnover, and they will typically be elevated in patients with Paget disease.^14^

Radiographs alone may not be enough to confirm the diagnosis of Paget disease, but they are used as first-line imaging before advanced imaging is considered. Radiographs generally demonstrate characteristic findings that include radiolucent lesions in the early disease, sclerotic changes in later disease, bone thickening with cortical expansion, trabecular disorganization (e.g., thick coarse trabecular pattern), bowing of long bones, and sometimes enlargement of the skull.^15^^,^^16^ Bone scintigraphy (technetium-99 radionuclide bone scan) is a highly sensitive imaging modality for detecting the extent of disease activity, showing increased uptake in affected areas due to increased bone turnover. In select cases, magnetic resonance imaging (MRI) or CT scan can be used to differentiate Paget disease from malignancy and for evaluating complications.^14^^,^^16^

The patient's radiographs and laboratory results are most consistent with Paget disease of bone. The physical exam demonstrates mild bowing and enlargement of the affected bone without signs of infection or acute fracture. X-ray findings show classic features, including mixed radiolucent and sclerotic changes, coarse trabecular thickening with cortical expansion, and deformity of the humeral diaphysis, all of which are findings associated with disorganized bone remodeling. The patient's BSAP is elevated, and collagen turnover markers such as urinary hydroxyproline and N- and C-terminal telopeptides are elevated, indicating accelerated bone turnover typical of Paget disease. Together, the clinical picture makes Paget disease of bone the most likely diagnosis.

### What are the signs and symptoms of Paget disease of bone?

In approximately 70–90% of Paget disease cases, the patient is asymptomatic.^12^

The most common symptom of Paget disease is bone pain, which may be related to microfractures, free nerve ending stimulation by dilated blood vessels, or weight-bearing in weakened bones.^17^ While bone pain is the most common presenting feature, other presentations include bone deformity, bowing of long bones (“saber shin”), enlarged skull, deafness, and pathological chalk-stick fractures.^13^^,^^18^ Patients may also be asymptomatic, and the only diagnostic clues are often rising serum ALP or incidental finding of characteristic lesions by radiographic findings.^13^ The patient may present with cranial nerve deficits, impaired hearing, headaches, and leonine facies. Other signs include cauda equina syndrome, nerve root compression, and spinal stenosis.^10^

Paget disease most commonly affects the pelvis, skull, vertebral column, and long bones of the lower extremities.^19^ Monostotic Paget affects only one bone and makes up 26.8% of the cases while polyostotic Paget affects two or more bones and makes up 73.2% of the cases.^20^

Metastatic carcinoma to bone presents with radiolucent and radiodense changes on imaging and occasionally with elevated ALP levels, which are both also found in Paget disease of bone.^21^ However, metastatic carcinoma often has multifocal metastatic lesions involving the spine, ribs, pelvis, and ends of long bones.^22^^,^^23^ Bone scintigraphy in bone metastases shows a random distribution, whereas Paget disease follows a more structured pattern along the affected bone.^24^^,^^25^ This so-called “geographic pattern” of bone involvement is more common with Paget. Hypercalcemia is not uncommon with widespread metastatic carcinoma to bone as calcium is leached out of the bone during osteoclastic bone resorption.

Lastly, multiple myeloma can also present with bone pain, fractures, and radiolucent lesions.^26^ However, multiple myeloma has a key differentiating feature of a “punched out” radiolucent lesion, while Paget disease shows broader mixed radiolucent and sclerotic regions of bone involvement.^10^^,^^27^ In addition, multiple myeloma presents with a specific monoclonal protein in the serum and urine, and it has normal to lower levels of ALP due to the multiple myeloma primarily causing osteolytic bone lesions while increased ALP is known to be caused by osteoblastic bone lesions.^28^^,^^29^ Both metastatic carcinoma to bone and multiple myeloma can have alterations in CBC, most commonly a decreased hemoglobin level. Acute inflammatory proteins, like erythrocyte sedimentation rate (ESR) are frequently elevated with both multiple myeloma and occasionally with MBD.

## Diagnostic findings, Part 2

Due to the possibility of other anatomical regions being affected by Paget disease, further imaging was obtained. Bone scintigraphy was performed on an outpatient basis.

## Questions/discussion points, Part 2

### Describe what you see in the bone scintigraphy scan

The findings show markedly increased activity throughout the entire left humerus, with mild increased activity in the L5 vertebral body (Fig. 2), suggestive of polyfocal involvement and thus supports Paget disease of bone.

### What is the pelvic brim sign on X-ray and what causes its appearance in Paget?

Paget disease of bone demonstrates characteristic radiographic features that reflect its disorganized pattern of accelerated bone turnover. Early lesions demonstrate well-demarcated radiolucent areas, such as the “blade of grass” or “flame-shaped” areas, caused by elevated osteoclastic activity.^10^ As the disease progresses, X-rays show a combination of coarse trabeculae and cortical thickening.^10^ In the advanced stages, bones become enlarged, while corticomedullary differentiation is often lost.^10^ These changes may involve the pelvis, femur, spine, skull, or humerus, and bone expansion with disorganized trabeculation is a hallmark feature of Paget disease of bone.^10^

If the Paget disease of bone affects the pelvis, the classic radiographic feature is the “pelvic brim sign.”^30^ It appears on X-ray as a thickened, dense, and radiopaque line along the pelvic brim, most prominently about the iliopectineal line.^30^ This occurs due to marked cortical thickening and sclerosis produced by Pagetic bone remodeling.^30^ The excessive bone formation leads to an accentuated, brightened outline of the pelvic brim.^30^ Its presence is considered highly suggestive of Paget disease and diagnostically valuable in helping differentiate Paget disease of bone from other disorders.^30^

### Describe the evolution of Paget disease using a 3-phase construct to better understand the pathology

Paget disease of bone is associated with a high rate of bone remodeling; increased receptor activator of nuclear factor-κB (RANK) ligand-RANK activity, leads to increased NF-κB signaling, increased osteoclast activity, increased osteoblast activity, and formation of disorganized and immature (woven) bone.^31^ Paget disease of bone is driven by abnormal osteoclast signaling from enhanced NF-κB activity, whereby the molecular alterations promote the hallmark accelerated bone resorption followed by disorganized osteoblastic repair.^32^ The best-established molecular defect is a mutation in the sequestosome 1 (*SQSTM1*) gene (which produces the p62 protein, so named due to its 62 kDa molecular weight), which leads to constitutive activation of NF-κB and excessive osteoclast differentiation and survival.^32^

**Fig. 3:**
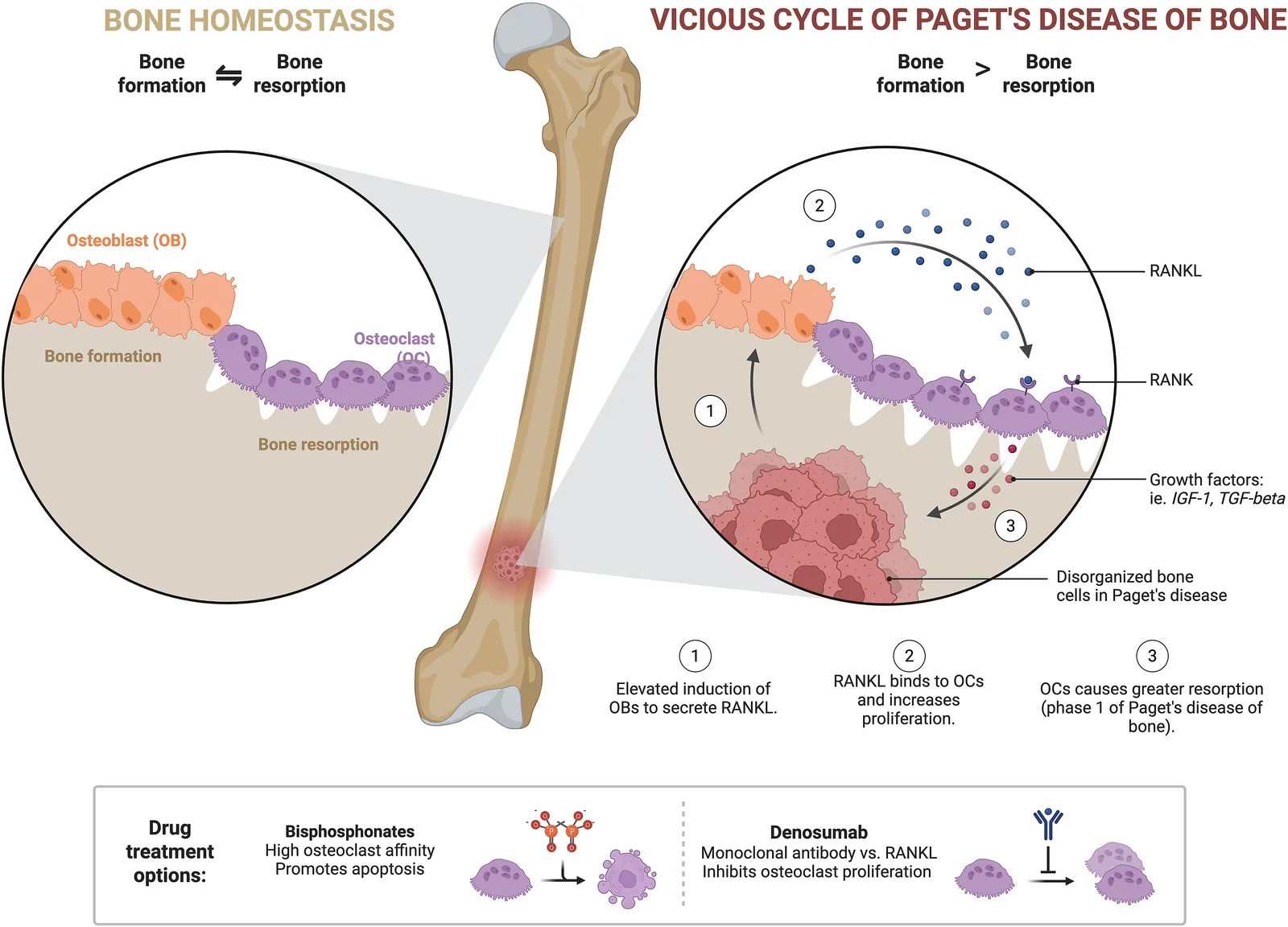
Normal bone remodeling (left) and aberrant remodeling in the early phase of Paget disease of bone (right). Created in BioRender.com by JJL.

Bone remodeling in Paget disease follows a progression (Fig. 3). The initial phase of Paget disease is the “hot” or osteoclastic resorptive (lytic or radiolucent) phase. An increased number of osteoclasts appear in the bone, and increased osteoclastic activity leads to increased rates of bone resorption.^17^ On radiographs, there is a characteristic flame- or wedge-shaped lysis of the bone cortex, which may mimic a tumor.^10^ On histology, there is widespread osteolysis, with marked osteoclastic resorption, marrow fibrosis, and dilation of marrow sinusoids.^17^ The initial osteoclastic lytic phase is triggered by overactive osteoclasts, which resorb bone at an accelerated rate, and the excessive bone breakdown creates regions of weakened bone.^17^

The next stage in progression is the mixed phase of osteoblastic and osteoclastic activity (the mixed lytic and blastic phase of the construct).^17^ Increased osteoclastic activity is accompanied by an increased number of osteoblasts, which infiltrate the lacunae, increasing rates of bone formation with haphazardly laid collagen fibers to form abnormally hypervascular woven bone.^14^ On radiographs, bones are larger than normal, with the cortex thickened, and the accentuation of the coarse cancellous bone makes the bone appear enlarged.^10^ In the vertebral bodies, the cortices and endplates become greatly exaggerated compared to the coarse cancellous bone of the vertebral body.^17^ On histology, there is evidence of both increased osteoclastic and osteoblastic activity.^17^ In response to the rapid bone loss from overactive osteoclasts, the osteoblasts compensate by forming new bone, marking the mixed osteoclastic-osteoblastic phase.^15^^,^^16^ The process is disorganized, leading to structurally abnormal bone with a woven pattern.^15^^,^^16^ The haphazard deposition leads to bone that is dense but weak.^15^^,^^16^

Subsequently, the “cold” or burned-out stage (sclerotic phase), in which osteoblastic activity overtakes osteoclastic activity, leads to the formation of dense, sclerotic bone.^16^^,^^17^ Radiographs shows thickened, disorganized, and chaotic bone structure, and on histology, there is little cellular activity.^17^ Hypercellularity is diminished as a sclerotic, less vascular mosaic pattern forms, and bone cell activity is greatly reduced.^15^

If Paget disease of bone progresses through its three stages, it does so sequentially due to the abnormal regulation of bone remodeling, and each phase is a direct consequence of the disruptions caused in the previous one.^15^ Over time, the osteoclastic activity will decline, leaving behind sclerotic bone lacking proper organization, indicative of the last stage in the evolution of Paget disease.^15^ The remodeling imbalance of bone formation and resorption results in a permanently altered bone architecture, making it prone to fractures and deformities.^16^

### What is the incidence of Paget disease of bone?

The incidence of Paget disease of bone has been declining over the past few decades, possibly due to reduced environmental triggers, with a possible decline in viral infections that are hypothesized to contribute to the development of the disease.^15^ While the viral cause of Paget disease of bone is not fully understood, it is hypothesized that paramyxoviruses, such as measles, canine distemper viruses, or respiratory syncytial viruses are causes of Paget disease.^15^

There are several risk factors that influence the incidence of Paget disease. Age greater than 50 years is associated with an increased incidence.^14^ There is a slight male predominance in prevalence, and it affects 1%–8% of the general population.^33^^,^^34^

### What complications are associated with Paget disease of bone?

Due to the structurally weak and disorganized bone, Paget disease can lead to several complications. The severity of the complications varies depending on the extent and location of the disease.

Skeletal complications can arise due to abnormal bone growth. They include pathologic fractures, bone deformities, and persistent and deep aching bone pain often due to microfractures.^35^ Bowing of weight-bearing bones can also occur, and the complications commonly affect the femur, tibia, and vertebrae because of axial loading.^14^^,^^15^

Neurological complications can occur because of compression from thickened bone.^14^, ^15^, ^16^ The bone can compress cranial nerves due to skull involvement, causing symptoms of nerve compression, including tinnitus or deafness (compression of cranial nerve eight, the vestibulocochlear nerve), and vertebral involvement can lead to spinal cord compression, leading to paresthesia and weakness.^14^, ^15^, ^16^ If the thickening of the skull is severe, it can obstruct cerebrospinal fluid flow, leading to obstructive hydrocephalus requiring neurosurgical intervention in severe cases.^15^

In patients with extensive Paget disease, though it is an exceedingly rare complication, the disease can lead to high output heart failure.^36^ The bones become hypervascular due to increased blood flow within the abnormal bone matrix, causing the formation of an excessive and disorganized network of blood vessels within the affected bone. Patients are found to have decreased peripheral vascular resistance and increased stroke volume, leading to possible high-output heart failure (left ventricular output >65%).^36^ Additionally, the increased vascularity of bone can lead to vascular steal syndrome due to the higher blood flow demand of Pagetic bone, causing blood to be diverted away from nearby neural tissue, which causes ischemia of the spinal cord, leading to myelopathy or worse, paraplegia.^16^

Rarely, malignant transformation of the Pagetic bone can occur, leading to Paget osteosarcoma.^15^^,^^16^ This occurs in less than 1% of cases but has a poor prognosis including roughly a 25% five-year survival rate in cases of metastatic disease, and clinical features include sudden worsening of bone pain, rapidly enlarging bone mass, and high ALP levels with progressive radiolucent bone lesions in pre-existing sclerotic bone on imaging.^15^^,^^16^

Patients with Paget disease have significantly increased fracture risk due to the abnormal structure and biomechanics of Pagetic bone.^37^ Although Pagetic bone is often thickened, it is also weaker and more brittle, making it prone to pathologic fractures, and the haphazardly arranged collagen and cement lines of the disorganized bone do not provide the same strength as normal lamellar bone.^37^ Pagetic bone is prone to transverse or stress fractures, especially within the weight-bearing bones.^37^ Chalk-stick fractures, which are transverse breaks across long bones, are a hallmark of Paget disease.^38^

The relative risk of fractures in Paget disease is estimated to be 3–5 times higher than in individuals without Paget disease, and up to 10–20% of Paget patients will experience at least one fracture in their lifetime.^37^ The risk is higher in polyostotic disease and in patients with severe bowing deformities.^39^

Recall that, in the early stages of Paget disease, osteoclasts resorb bone aggressively. In response to this bone loss, osteoblasts overproduce new bone in a disorganized “mosaic” or “jigsaw” pattern, which thickens the bone.^17^ This leads to the expansion of the skull diploe, particularly in the frontal and occipital regions. The diploe is the spongy cancellous bone between the inner and outer cortical layers of the skull. The diploe becomes wider and more vascular, increasing skull thickness, and this process gives rise to the classic “Tam o’ Shanter sign”, which means an “enlarged skull with a hat that no longer fits”.^40^ Patients will notice that their hats no longer fit due to progressive skull thickening.

## Diagnostic findings, Part 3

Biopsy of the patient's humerus was obtained by interventional radiology to confirm the diagnosis and exclude other bone-forming etiologies because of several months of persistent pain and new-onset night pain. Bone biopsy is not routinely recommended or performed during the late stage of Paget disease.

## Questions/discussion points, Part 3

### Describe the pathology based on the humerus biopsy and its corresponding 3-construct Paget disease stage

**Fig. 4:**
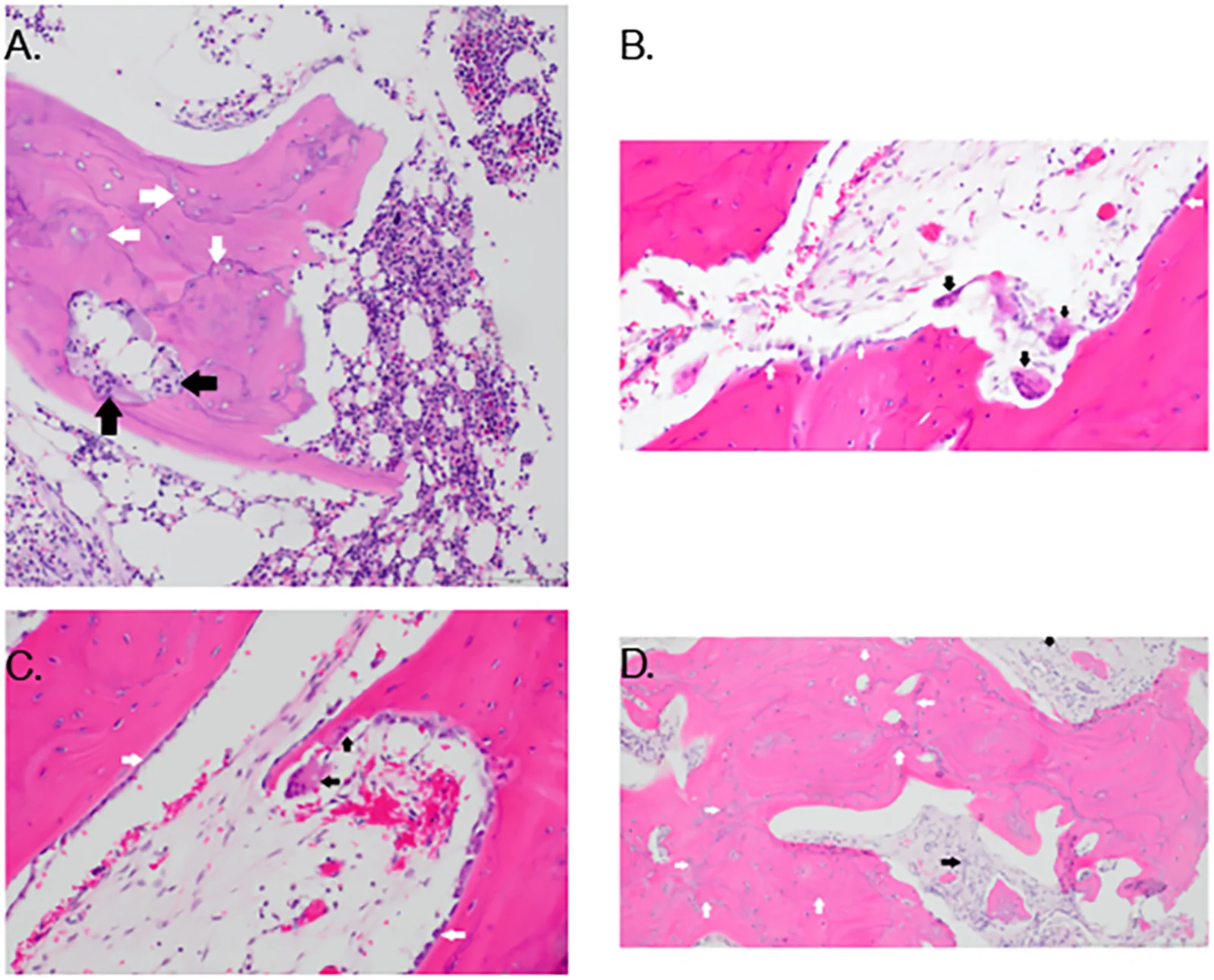
**(A)** Low-power microphotograph showing irregular cement lines (white arrows) in a mosaic pattern and increased osteoclast activity forming resorption pits (black arrows) (Hematoxylin & Eosin, 10X). **(B)** High-power microphotograph demonstrating increased osteoblastic activity (white arrows) and osteoclastic activity (black arrows) (Hematoxylin & Eosin, 20X). **(C)** Low-power microphotograph showing thickened, sclerotic bone with a mosaic pattern of cement lines (white arrows) and marrow fibrosis (black arrows) (Hematoxylin & Eosin, 10X). **(D)** High-power microphotograph showing numerous cement lines (white arrows) due to rapid bone turnover, giving the bone a characteristic appearance jigsaw/mosaic pattern, and marrow fibrosis (black arrows) (Hematoxylin & Eosin, 20X). Created in BioRender.com by JJL.

The left humerus biopsy demonstrated characteristic histological findings of Paget disease of bone. The hematoxylin and eosin (H&E) findings are summarized below in Fig. 4.

Figure 4A demonstrates a low-power microphotograph showing irregular cement lines (white arrows) in a mosaic pattern and increased osteoclast activity forming resorption pits (black arrows) representing early Paget disease. Fig. 4B is a high-power microphotograph demonstrating increased osteoblastic activity (white arrows) and osteoclastic activity (black arrows), representing a mixed Paget disease stage. Fig. 4C shows a low-power microphotograph showing thickened, sclerotic bone with a mosaic pattern of cement lines (white arrows) and marrow fibrosis (black arrows), consistent with the late stage of Paget disease. Lastly, Fig. 4D demonstrates a low-power microphotograph with thickened, sclerotic bone, a mosaic pattern of cement lines (white arrows), and marrow fibrosis (black arrows) supporting a mixed stage.

### What do normal mature bone, immature bone, and Paget bone look like microscopically?

Normal bone appears in two primary forms: woven immature bone and lamellar mature bone.^41^

Woven bone has an irregular, disorganized collagen fiber arrangement with high osteocyte density, and the bone matrix is poorly mineralized. Woven bone can be found in fetal skeletons, sites of fracture healing, and pathologic conditions.^42^ Its function is to serve as a temporary structure before it is replaced by lamellar bone.^43^

Lamellar bone has an organized parallel or concentric arrangement of collagen fibers with fewer osteocytes compared to woven bone, and this type is found in cortical and trabecular bone of the adult skeleton. It is stronger and more resilient than woven bone.^44^

Pagetic bone is highly disorganized and structurally weak, with hallmark histological changes across three stages.^12^ The early lytic phase with increased osteoclastic activity shows large, multinucleated osteoclasts with excessive nuclei (>10 per cell) and bone resorption pits due to rapid bone breakdown.^9^^,^^31^ During the mixed osteoclastic-osteoblastic phase, intermixed woven and lamellar bone can be found along with irregular cement lines forming a disorganized mosaic pattern, and marrow spaces become increasingly filled with fibrovascular tissue.^45^ The late sclerotic phase shows thickened trabeculae and cortex due to excessive bone deposition with a persistent mosaic pattern due to repeated cycles of bone remodeling. However, despite the thickened bone, it remains poorly mineralized.^30^

### What is the recommended treatment for Paget disease of bone?

The goal of treatment in Paget disease is to reduce bone turnover, relieve symptoms, and prevent complications. Treatment is typically reserved for symptomatic patients or patients at risk of complications.^14^

The first-line therapy for Paget disease is bisphosphonates, which function to inhibit osteoclast activity, reducing excessive bone turnover and promoting normal bone remodeling.^46^ The mechanism of action is through inducing apoptosis of osteoclasts.^47^ Bisphosphonate drugs include zoledronic acid, alendronate, risedronate, pamidronate, and tiludronate. The preferred drug is zoledronic acid 5 mg through IV infusion as a single infusion annually due to its strong potency, long-lasting suppression of bone turnover, and single-dose infusion.^48^^,^^49^ Contraindications for bisphosphonates include severe kidney disease with a GFR (glomerular filtration rate) less than 30–35 mL/min, hypocalcemia, and esophageal disorders for oral bisphosphonates. If the patient has hypocalcemia, calcium or vitamin D can be used to correct serum calcium levels during bisphosphonate therapy.^50^ Sitting upright for a minimum of 30 minutes after taking oral bisphosphonates may also decrease the risk of esophagus mucosal injury.

The alternative therapy is calcitonin in nasal spray or injection form, which can modestly reduce bone turnover but is less effective than bisphosphonates. It is used only if bisphosphonates are contraindicated.^13^

For symptom management, non-steroidal anti-inflammatory drugs (NSAIDs) can be used for the bone pain.^14^

For severe Paget disease cases, surgical management may be needed to treat complications. Indications for surgery include severe bone deformities, improperly healing fractures, osteoarthritis causing joint destruction, and nerve compression.^15^ Risks for surgery in Paget patients include excessive bleeding due to the hypervascular bone, delayed bone healing due to the disorganized bone structure, and a higher risk of fractures around implants.^15^ Preoperative risk stratification can include a cardiology consultation, during which the patient may require an echocardiogram to assess cardiac output. Anesthesia may utilize the results of the echocardiogram to optimize anesthesia and lower the patient's risk of an intra-operative incident.

## Teaching points

• Paget disease of bone causes formation of disorganized woven bone due to increased osteoclastic and osteoblastic activity.
• The evolution of Paget disease of bone consists of the initial osteoclastic radiolucent phase, the mixed radiolucent and sclerotic phase, and the late burnt-out sclerotic phase, which follow in sequential order.
• Initially, Paget disease is marked by increased osteoclastic activity leading to accelerated bone resorption, which creates regions of weakened bone and marrow fibrosis.
• As Paget disease progresses, osteoblastic activity increases, which is coupled with the increased osteoclastic activity, leading to the formation of abnormally disorganized bone with a woven pattern.
• Eventually, osteoblastic activity increases more than the osteoclastic activity, forming thickened and disordered sclerotic bones with reduced cellular activity.
• Paget disease of bone can lead to skeletal, neurologic, and vascular complications due to disorganized and structurally weak bone remodeling, increasing the risk of fracture, nerve compression, and high-output heart failure. Severe cases may involve deformities, hydrocephalus, and rare malignant transformation to osteosarcoma.

## Funding

The article processing fee for this article was funded by an Open Access Award given by the Society of ‘67, which supports the mission of the Association for Academic Pathology to produce the next generation of outstanding investigators and educational scholars in the field of pathology. This award helps to promote the publication of high-quality original scholarship in *Academic Pathology* by authors at an early stage of academic development.

## Declaration of competing interest

The authors declare that they have no known competing financial interests or personal relationships that could have appeared to influence the work reported in this paper.
